# Development of Bionic Semicircular Canals and the Sensation of Angular Acceleration

**DOI:** 10.3390/bioengineering9050180

**Published:** 2022-04-20

**Authors:** Zhi Wang, Shien Lu, Xianjin Wang, Yuhang Chen, Junjie Gong, Yani Jiang, Yixiang Bian

**Affiliations:** Department of Mechanical and Electrical Engineering, College of Mechanical Engineering, Yangzhou University, Yangzhou 225012, China; laughlin222222@163.com (Z.W.); huhu1232022@163.com (S.L.); huyue0131@163.com (X.W.); ruyi1311@126.com (Y.C.); jjgong@yzu.edu.cn (J.G.)

**Keywords:** human semicircular canals (HSCs), three-dimensional bionic semicircular canal (3-BSC), one-dimensional bionic semicircular canal (1-BSC), straight semicircular canal, bionic ampulla (BA), symmetric electrode metal core PVDF (polyvinylidene difluoride) fiber (SMPF)

## Abstract

To study the sensing process of the human semicircular canals (HSCs) during head rotation, which is difficult to directly measure due to physiological reasons. A 1-BSC (one-dimensional bionic semicircular canal) and 3-BSC were prepared with soft SMPFs (symmetric electrode metal core polyvinylidene difluoride fibers), which could sense deformations similar to human sensory cells. Based on these models, experiments were carried out to study the principle of the HSCs. Deformations of the bionic ampulla (BA) depended on the angular acceleration. Gravity had a strong influence on the deformation of the BA in the vertical plane. When the 3-BSC was subjected to angular acceleration around one of its centerlines, the three BAs all deformed. The deformation of the BAs was linearly related to the angular acceleration. The deformation of the BA in the main semicircular canal was exactly three times that of the other two BAs.

## 1. Introduction

The vestibular system is an important organ in the human body, maintaining stable vision and body balance in space and including three pairs of semicircular canals and two pairs of otolith organs that are symmetrically distributed in the inner ear on both sides of the head [1]. The human semicircular canals (HSCs) can sense the angular acceleration of the head, while the macular organs can sense its linear acceleration [2]. The HSCs on two sides of the head have the same shape. Each HSC comprises a ring that is geometrically positioned approximately orthogonal to the other two HSCs [3]. All three HSCs are filled with endolymph and communicate with the utricle. In each HSC, there is a sickle-shaped crest in the enlarged ampulla. The sensory hair cells are hidden in the bottom of the crista ampulla, and their ciliary bundles insert into the top of the gelatinous cupula above the crest [4]. When the human head rotates, the walls of the HSC also rotate. However, due to inertia, the rotation speed of the endolymph in the HSC is smaller than that of the wall. When the rotational inertia of the endolymph acts on the cupula, this deforms the cupula, resulting in bending deformation of the ciliary bundles of the sensory hair cells. At the same time, sensory hair cells produce nerve signals that are transmitted to the vestibular center and brain through the ascending nervous system [5]. According to the nerve signal, the brain can sense the magnitude and direction of the angular acceleration of the human head [6,7].

The capability of the HSCs to perceive the angular acceleration of the head depends on the deformability of the cupula in the ampulla. The deformation of the cupula is caused by the endolymph flow in the HSC [8]. Therefore, to fully understand the perception mechanism of the HSC, it is necessary to study the deflection of the cupula due to endolymph movement when the HSCs rotate. Some researchers have studied the deformation characteristics of the cupula by performing experiments on animals such as bullfrogs [9] and rats [10]. Shen et al. established a model for a three-dimensional semicircular canal using the finite element method and carried out the modal analysis, excitation response analysis, and frequency characteristic analysis of the endolymph–cupula coupling system [11].

These typical biomechanical models have greatly promoted the study of the relationship between the structure and function of HSCs. However, it is difficult to directly determine the mechanical response of the HSCs using current technology, such as medical imaging [11,12]. This is because the vestibular system is located deep within the human head, the volume of the HSCs is small, and the structure is complex. Additionally, due to the limitations on human physiological conditions, many physical tests cannot be carried out; therefore, it is difficult to directly verify or effectively verify biomechanical theoretical models and the numerical models of the HSCs [13]. At present, the status of the vestibular function is mainly judged according to the vestibulo-ocular reflex (VOR), vestibular spinal reflex, and vestibular autonomic nerve reflex [2]. All of these factors have hampered our understanding of vestibular pathology and the development of corresponding treatment methods [14,15].

Recently, a few physical models have been fabricated and used to carry out experiments that are difficult to perform in the human body. Dominik et al. designed and prepared a physical one-dimensional semicircular canal model. In their model, an elastic material, viscous liquid, and small steel balls were used to imitate the cupula, endolymph, and otoliths, respectively. The motion trajectories of the otolith in the HSCs during benign paroxysmal positional vertigo (BPPV) were studied by tracking the motion trajectory of the small steel balls in the physical model using an electronic camera [16,17]. This model itself had no perception function; thus, it was quite different from the HSCs. Mohammad et al. prepared a physical semicircular canal model in which a piezoresistive cantilever sensor was used to imitate sensory hair cells [18]. The model exhibited good sensing ability, as it could clearly sense sinusoidal vibration. In their recent research, a new semicircular canal model was prepared using a flexible cantilever sensor based on graphene nanosheets [19]. These two models exhibited good sensing ability; however, the flow process and flow field distribution were different from those in the HSCs because their shapes were different. The above-mentioned studies indicated that it was a feasible to study the HSCC through physical models. A bionic semicircular canal (BSC) model, with a structure and perception process similar to those of the HSCC, can be used to carry out experiments in vitro and to help people to understand the function of HSCs [20].

The SMPF (symmetric electrode metal core PVDF (polyvinylidene difluoride)) fiber sensor that was successfully fabricated in our previous work is a new type of flexible sensor [21], having a simple structure, small volume, and good flexibility [22]. Therefore, in this study, imitating the sensing function of the sensory cilia, a bionic ampulla (BA) was designed and fabricated by embedding an SMPF into silicone rubber. The shells of the semicircular canal were separately prepared using 3D printing method. Then, a bionic 1-BSC (one-dimensional bionic semicircular canal) and the 3-BSC (three-dimensional bionic semicircular canal), with a ratio of 1:1 to the HSCs, were obtained by assembling the BA in the shells and then filling the shells with deionized water to imitate the endolymph in HSCs. Due to their bionic structure and function, the 1-BSCs and 3-BSCs are not just bionic units of human semicircular canals but also bionic organs. Finally, various angular acceleration sensing experiments were carried out based on the 1-BSC and 3-BSC, and the relationship between the structure of the HSCs and their function was explored.

## 2. Materials and Methods

### 2.1. Materials 12

The PVDF 6008 particles were purchased from Solvay Corporation. The metal core was composed of molybdenum wire (Φ 0.11 mm, Nanjing Juxin Tungsten Molybdenum Co., Ltd., Nanjing, China). The surface electrodes were composed of conductive silver paste (3706, Shenzhen Sinwe Electronic Material Co., Ltd., Shenzhen, China). The cupula was made of silicone rubber (E610, Shenzhen Hongye Technology Co., Ltd., Shenzhen, China). The 3D printing materials were standard resins (A01, Shenzhen Chuangxiang 3D Technology Co., Ltd., Shenzhen, China).

### 2.2. Fabrication Methods

#### 2.2.1. Preparation of the SMPF

**Figure 1:**
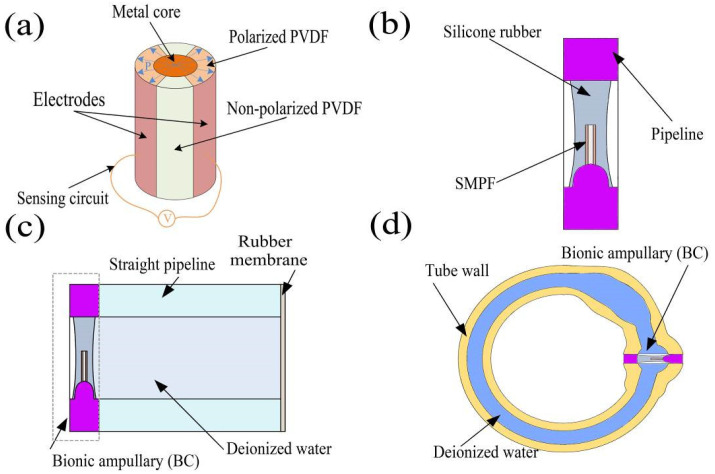
Diagrams of the structure of an (**a**) SMPF and circuit, (**b**) bionic ampulla (BA), (**c**) straight semicircular canal, and (**d**) one-dimensional bionic semicircular canal (1-BSC).

The SMPF was prepared according to the procedure described in our previous study [22]. Briefly, the PVDF particles were heated to 170 °C to a molten state. Then, the molten PVDF was extruded together with a tungsten wire. A fiber embryo was obtained after air cooling, as shown in Figure 1a. Two symmetrical thin metal layers were coated on the longitudinal surface as surface electrodes. Then, the fiber embryo was put into silicone oil at 130 °C and a 500 V voltage was applied to its surface electrodes for 1 h. An SMPF sensor was obtained after cleaning and connecting the electrodes, and a diagram of its structure was shown in Figure 1a.

#### 2.2.2. Preparation of the BA

Based on the structure of the ampulla in the human semicircular canal [23,24], the BA shell was prepared by 3D printing. An SMPF was placed in the mold, and its root was fixed on the BA shell. Then, medical silicone rubber solution was injected into a mold, and a BA was successfully fabricated after the solution was solidified. A diagram of the structure of BA is shown in Figure 1b.

#### 2.2.3. Preparation of the Straight Semicircular Canal

A BA was installed on one end of a straight pipeline prepared by 3D printing, and the other end was sealed with a rubber membrane. The straight pipeline was filled with deionized water and was sealed, yielding a straight semicircular canal. A diagram of its structure is shown in Figure 1c. One side of the BA was surrounded by deionized water, and the other side was exposed to air.

#### 2.2.4. Preparation of the 1-BSC and 3BSC

The shells of the 1-BSC and 3-BSC were prepared using the 3D printing method. A BA was installed on the ampulla and the shell was filled with deionized water and sealed, yielding a 1-BSC. Three BAs were separately installed on the three ampullas, yielding a 3BSC. The 1-BSC and 3BSC have ratios of 1:1 to the HSCC, respectively.

### 2.3. The Experimental Methods

#### 2.3.1. The Straight Pipeline Semicircular Canal Experiment

A piezoelectric stack driver was used to apply pressure to the elastic rubber membrane at one end of the straight pipeline semicircular canal. Then, the deionized water in the pipeline was squeezed by the rubber membrane and the pressure was transported to the BA at the other end of the straight pipeline, which caused concave and convex deformation of the SMPF in the cupula, yielding electric charges. The electric charges of the SMPF were converted into voltage through a charge amplifier to characterize the bending deformation of the SMPF.

#### 2.3.2. 1-BSC Experiment

A system was built to carry out swing experiments on the 1-BSC. The 1-BSC was fixed on a worktable, and an electromagnetic exciter was used to drive the 1-BSC to perform an impact swing, sinusoidal swing, and square wave swing around its axis line through a gear rack structure. The displacement of the electromagnetic exciter was measured using a laser displacement sensor, and then the displacement was converted into the angular displacement and angular acceleration of the 1-BSC through calculations. The worktable was separately placed in the horizontal plane and vertical plane to study the ability of the 1-BSC to sense the angular acceleration and the influence of gravity on it.

#### 2.3.3. 3-BSC Experiment

The worktable was fixed in the horizontal plane, and one of the three semicircular canals in 3-BSC was fixed parallel to the worktable, which was called the main semicircular canal. An electromagnetic exciter was also used to drive the 3-BSC to swing. The center of rotation fell on the center point of the ring of the main semicircular canal. Then, the sensing angular acceleration experiment of the main semicircular canal was carried out. After the experiment was over, the other semicircular canal was fixed as the main semicircular canal and the angular acceleration sensing experiment was continued.

#### 2.3.4. Experimental Data Collection

The electric charges on the SMPF were simultaneously input into the computer through the channel of the data acquisition card. The displacements of the subject were measured using a laser displacement sensor and were input into the computer through a signal acquisition card.

## 3. Results and Discussion

### 3.1. The Micro- and Macrostructures of Production

**Figure 2:**
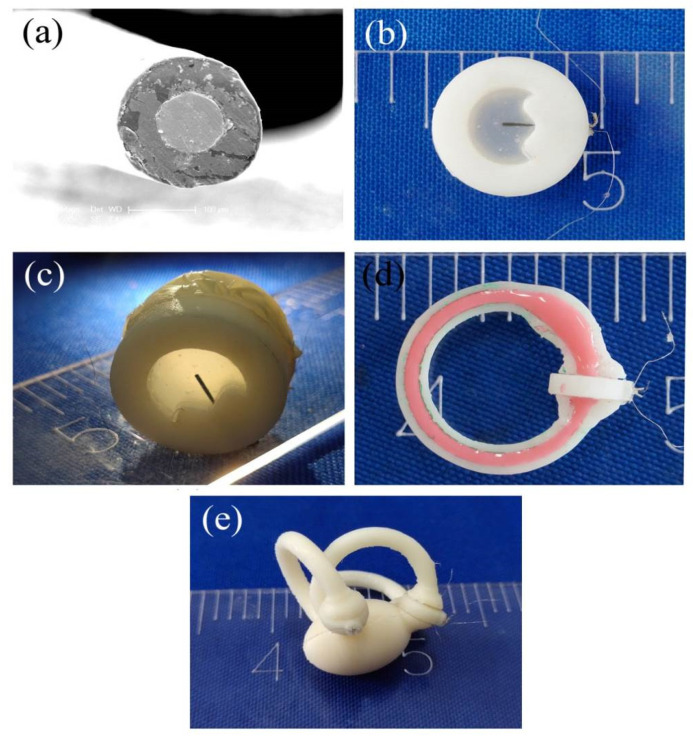
(**a**) An SEM (scanning electron microscope) image of the cross-section of the SMPF, (**b**) a photograph of a BA, (**c**) a photograph of the straight semicircular canal, (**d**) a photograph of the section of the 1-BSC, and (**e**) a photograph of the 3-BSC.

PVDF is an organic piezoelectric material with direct electromechanical conversion performance capability. The SMPF sensor contained two surface symmetric electrodes and one metal core PVDF fiber. The metal core was located in the center, and the PVDF layer with uniform thickness was wrapped around the metal core. An SEM (scanning electron microscope) image of the cross-section of the SMPF is shown in Figure 2a. The diameter of the metal core was 100 μm, and the outer diameter of the PVDF layer was 180 µm. Two thin metal layers were coated symmetrically on the surface of the PVDF cylinder along the longitudinal direction and were used as surface electrodes. Part of the PVDF layer covered with the surface electrodes was polarized along the diameter direction and exhibited a piezoelectric effect. However, the rest of the PVDF layer not covered with the surface electrodes and was not polarized, meaning it exhibited no piezoelectric effect. Due to the piezoelectric effect and the symmetrical structure of the PVDF layer, sensing charges of equal amplitude and opposite polarity were generated on the two surface electrodes when the SMPF sensor was bent. According to the values of the sensing charges in the sensing circuit, the direction and degree of the bending deformation of the SMPF could be calculated. The SMPF sensor is small and has good flexibility and the ability to sense bending deformation; therefore, it is very suitable for imitating the hair cells in the human vestibule that sense concave and convex deformations of the cupula.

A photograph of the BA is shown in Figure 2b. In the BA, silicone rubber was used to imitate the cupula in the human semicircular canal, and an SMPF was embedded in silicone rubber to prepare the BA. Both silicone rubber and SMPF have good flexibility and certain elasticity; therefore, the BA could produce concave and convex deformations under liquid pressure, and it could return to its original shape after the pressure was removed. The BA exhibited mechanical performance similar to that of the cupula in the human semicircular canal. The SMPF could sense the direction and amplitude of the concave and convex deformation of the bionic cupula. Therefore, the BA had a perception function similar to sensory hair cells in the human semicircular canal.

The photographs of the fabricated straight pipeline semicircular canal, 1-BSC, and 3-BSC are separately shown in Figure 2c–e. The 1-BSC and 3-BSC imitate the complex structure of human semicircular canals and also their function; that is, the function of sensing angular acceleration. Therefore, they are both bionic systems.

### 3.2. The Results of the Straight Semicircular Canal Experiments

**Figure 3:**
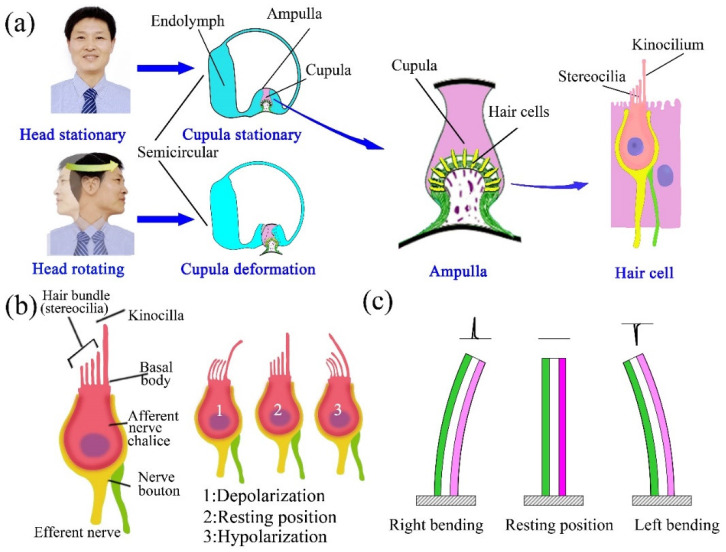
(**a**) A diagram of the HSCs, ampulla, and sensory hair cells. (**b**) A diagram of a hair cell to show its cilia incline direction through its state of depolarization or hyperpolarization. (**c**) A diagram of the SMPF to show its deformation direction through the generation of positive or negative charge polarity.

In the HSCs, sensing organs are located in the ampulla, which are sensory hair cells embedded in the cupula, as shown in Figure 3a. The cilia on the top of the sensory hair cells can remain upright in a static state, as shown in Figure 3a,b. Under angular acceleration of the head, the hair cells experience depolarization and cause the HSCC to be in an exciting status when stereocilia incline towards kinocilia due to the inertia force of the endolymph, while they experience hyperpolarization and cause the HSCC to be in an inhibited state when the incline direction is opposite [16,17]. The directions and amplitudes of the incline can be transmitted to the brain by the HSCs according to status [25]. A diagram of the SMPF sensing function is shown in Figure 3c. When the SMPF bends, charges can be generated on it due to the piezoelectric effect of the PVDF, and the polarity of the charge generated when the SMPF bends to the left is opposite to that to right. The directions and amplitudes of the bend deformation can be obtained according to the polarity and amount of charge. Therefore, in theory, the function of the SMPF is similar to that of the hair cells, meaning they both can sense the directions and amplitudes of the bend.

**Figure 4:**
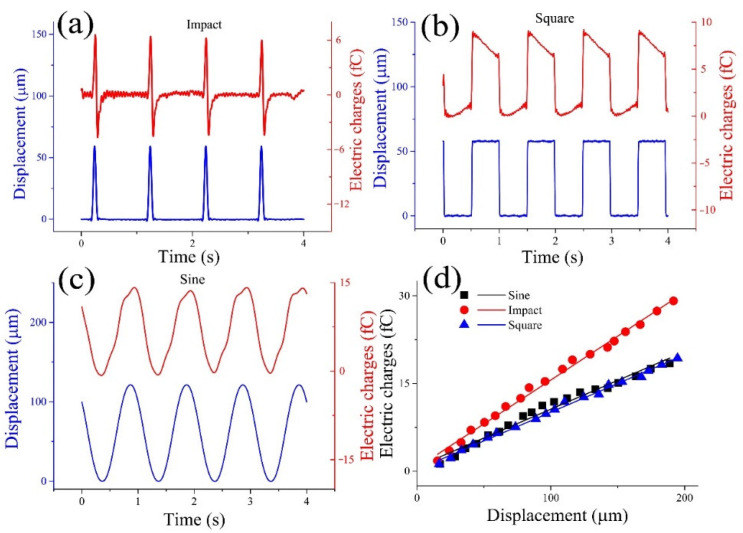
Deformation experiment of the cupula, whereby (**a**) the cupula was separately subject to an impact vibration, (**b**) a square vibration, and (**c**) a sinusoidal vibration. (**d**) The relationship between the amplitude of cupula deformation and the amplitude of output electric charges.

To study the relationship between the SMPF output signal and the concave and convex deformations on the cupula of the BA, straight pipeline experiments were carried out. As shown in Figure 4a, when the cupula of the BA was deformed by impact vibration, the electric charges also exhibited an impact waveform. The phase of the positive amplitude of the electric charge was the same as that of the cupula deformation, while the phase of the negative amplitude of the electric charge was different from that of the cupula deformation. This difference was caused by the inherent characteristics of the piezoelectric sensor. When the cupula of the BA was deformed by a square vibration, the waveform of the electric charges output by the SMPF was not square, as shown in Figure 4b. When the cupula of the BA underwent positive or negative step deformation, the value of the electric charges suddenly increased or decreased while its phase was the same as that of the variation. However, when the cupula remained continued to have the highest displacement, the electric charge value continued to fall; when the cupula continued to have the lowest displacement, the charge value continued to rise. This phenomenon was caused by the inherent characteristics of the piezoelectric sensor. As shown in Figure 4c, when the cupula was subjected to a sinusoidal vibration, the sensing signals of the SMPF also exhibited a sinusoidal waveform, while the phase was the same as that of the cupula deformation.

The relationship between the vibration displacement amplitude of the cupula of a BA and the amplitude of the electric charges output by the SMPF is shown in Figure 4d. The slope of the fitting line was defined as the sensing sensitivity. The sensitivity to the shock vibration was 0.15924 fc/μm, to the square vibration was 0.09826 fc/μm, and to the sinusoidal vibration was 0.09858 fc/μm. The sensitivity to the shock vibration was the strongest, while the sensitivity of the square vibration was basically the same as that of the sinusoidal vibration.

According to the above results, the type, amplitude, and frequency of the cupula deformation displacement of the BA can be derived based on the electric charges output by the SMPF and the inherent characteristics of the piezoelectric sensor. Through further processing of the data in the above experimental results, a linear relationship between the amplitude of the cupula deformation and that of the electric charges output by the SMPF was obtained, as shown in Figure 4d. This result occurred because when the BA was subjected to liquid pressure in the straight pipeline, whereby its cupula produced concave and convex deformations. According to the first kind of piezoelectric equation and the elastic membrane theory, the relationship among the electric charges output by the SMPF $Q_{1}$, the cupula center point displacement *H*, and the internal liquid pressure P can be described as follows (the specific derivation process of Equation (1) is shown in the Supplementary Materials):

(1) $$Q_{1}=K_{1}H-K_{2}P$$

where $K_{1}$ and $K_{2}$ are constants determined by various parameters of the BA. It can be seen that there was a linear relationship between the charge and the displacement. This meant that the displacement amplitude at the center point of the BA could be calculated. According to the electric charges output by the SMPF, the direction, type, frequency, phase, and amplitude of the concave and convex vibration deformation of the BA could be determined and calculated.

The results indicated that the BA not only had a structure similar to the ampulla in the human body but also exhibited a function similar to the ampulla; that is, it was able to sense the directions and amplitudes of deformation according to the polarity and amplitude of the output charge. Therefore, the semicircular canal physical models prepared with the BA in this paper also had similar sensing functions to the HSCs and could be used as intelligent tools for the study of HSCs.

### 3.3. The Results of the 1-BSC Experiments

When the 1-BSC was driven to swing around its centerline, the SMPF embedded in the BA could generate electric sensing charges. This was because when the 1-BSC was subjected to angular acceleration in a short time, rotational movement occurred in the canal and the BA. The liquid in the canal could rotate in the direction opposite to the canal wall in a short time due to inertia, resulting in pressure on the adjacent BA. Then, concave and convex deformation of the BA occurred, while bending deformation of the SMPF embedded in the BA occurred at the same time. Because of the piezoelectric effect, the SMPF could output electric charges. The electric charges output by the SMPF can be described as follows (the specific derivation process is shown in the Supplementary Materials):

(2) $$Q_{2}=K_{3}MR\alpha$$

where M is the liquid mass in the canal and R is the canal radius. It can be concluded that there was a linear relationship between the electric charge of the SMPF and the angular acceleration α of the 1-BSC.

**Figure 5:**
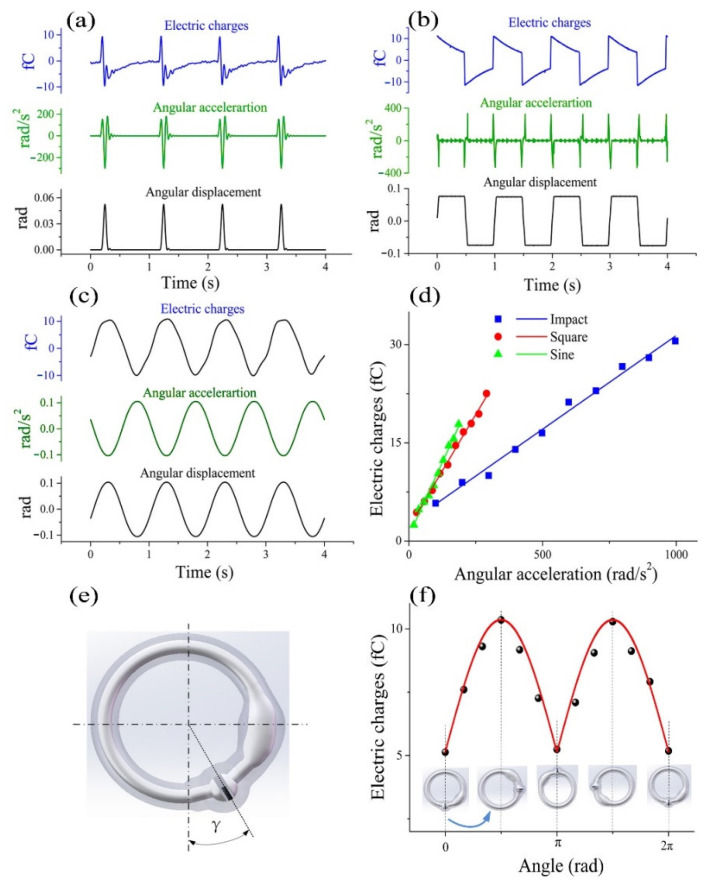
Experimental results for sensing angular acceleration using the 1-BSC: (**a**) the relationship between the amplitude of angular acceleration and the amplitude of output electric charges of the 1-BSC; (**b**) the 1-BSC sensed impact angular displacement; (**c**) the 1-BSC sensed square angular displacement; (**d**) the 1-BSC sensed sinusoidal angular displacement; (**e**) diagram of the angle *γ*; (**f**) the relationship between the amplitude of the electric charges output by the SMPF and the angle *γ*.

When the 1-BSC was subject to an impact swing, an impact angular acceleration occurred, as shown in Figure 5a. According to the electric charges output by the SMPF, the displacement of the cupula center of the BA exhibited an impact waveform that had the same phase as that of the impact swing, while the amplitude of the deformation displacement was linearly related to that of the angular acceleration. When the 1-BSC was subjected to a square swing, there were a series of impact angular accelerations on the 1-BSC at the same time, as shown in Figure 5b. According to the electric charges, the deformation on the center of the cupula had a square waveform, while the amplitude of the deformation displacement was linearly related to that of the angular acceleration. When the 1-BSC was subjected to a sinusoidal swing, there was a sinusoidal angular acceleration on it. The electric charges indicated that the displacement on the center of the cupula of the BA also exhibited a sinusoidal waveform, as shown in Figure 5c. Based on these results, it can be speculated that when the human head is subjected to an impact swing, a sinusoidal swing, or a square swing, the corresponding deformation of the cupula also exhibits an impact waveform, a sine waveform, or a square waveform, respectively.

When the 1-BSC was subjected to swings with three different waveforms, the relationships between the angular accelerations and the BA output electric charges were identified, as shown in Figure 5d. The frequency was 1 Hz and the amplitude range was from 0.5 to 5 mm. It can be observed that when the amplitude of the swing was the same, the order of the types of swing in terms of increasing angular acceleration was sine swing > square swing > shock swing. The angular acceleration of the shock swing was much higher than those of the other two kinds of swings.

In most theoretical models of endolymph flow in the human semicircular canal, the influence of gravity has been ignored [26,27,28,29]. To study the influence of gravity on the perception of the angular acceleration of the semicircular canal, the 1-BSC was placed in the vertical plane and the angle between the BA and the plumb line was defined as angle γ, as shown in Figure 5e. The 1-BSC was driven to swing around its own centerline in the vertical plane. Under impact swings with the same frequency and amplitude, when the BA was located at different γ angles, the displacement of the center of the cupula was very different. These results indicated that the displacement of the cupula was related not only to the angular acceleration but also to the angle γ. This occurred because when the 1-BSC swings around its centerline in the vertical plane with an angular acceleration α, the output electric charges of the SMPF can be calculated as follows (the specific derivation is shown in the supplemental materials):

(3) $$Q_{4}=K_{4}M\left(R\times \alpha +g\left|\sin\gamma \right|\right)$$

where g represents the gravity acceleration. It can be concluded that there was a sinusoidal relationship between the displacement of the cupula center and the angle γ. These results helped us to understand the effect of gravity on the perception of the angular acceleration of the human semicircular canal.

In the experiments, when the BA was located at different γ angles, the 1-BSC was subjected to a sinusoidal swing at the same frequency and amplitude. The relationship between the amplitude of the electric charges output by the SMPF and the angle γ is shown in Figure 5f. It can be observed that the electric charges output by the SMPF based on the angular acceleration were sinusoidal with γ. The experimental results verify Equation (3).

### 3.4. The Results of the 3-BSC Experiments

**Figure 6:**
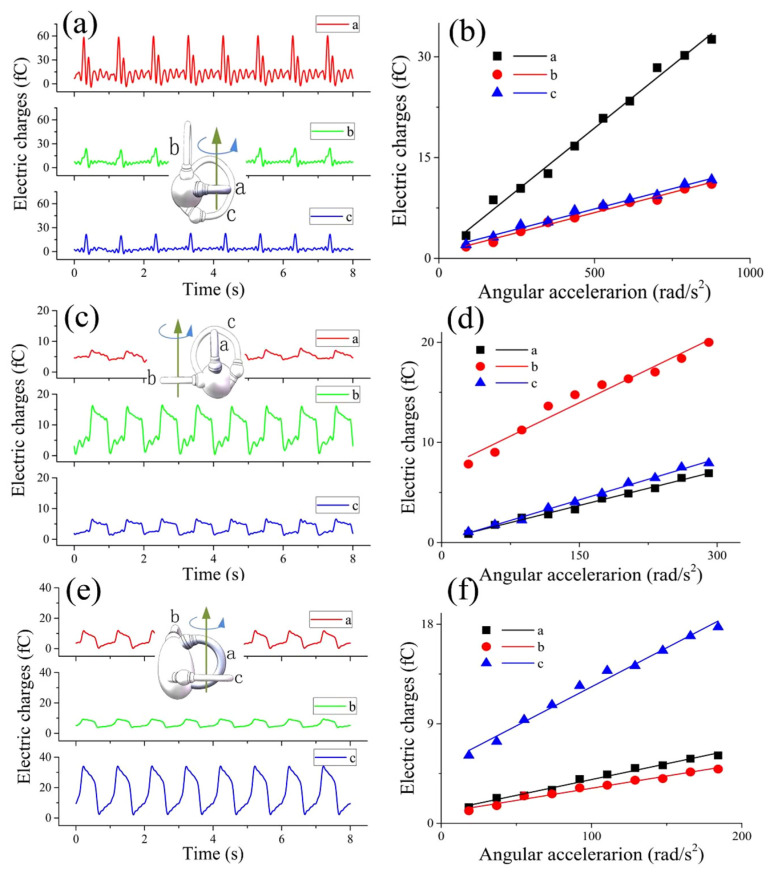
Experimental results for sensing angular acceleration using the 3-BSC: (**a**) the 3-BSC sensed impact angular displacement; (**b**) the relationship between the amplitude of impact angular acceleration and the amplitude of output electric charges of the 3-BSC; (**c**) the 3-BSC sensed square angular displacement; (**d**) the relationship between the amplitude of square wave angular acceleration and the amplitude of output electric charges of the 3-BSC; (**e**) the 3-BSC sensed sinusoidal angular displacement; (**f**) the relationship between the amplitude of sinusoidal angular acceleration and the amplitude of output electric charges of the 3-BSC.

For convenience, the horizontal semicircular canal, the posterior semicircular canal, and the anterior semicircular canal of the 3-BSC were separately labeled canals a#, b#, and c#. The output electric charges of the three semicircular canals when the 3-BSC was subjected to an impact swing around the centerline of the a# canal is shown in Figure 6a. It can be observed that the electric charges output by the three canals all exhibited impact waveforms and that the phases of the three electric charges were the same, which indicated that the displacements of the cupula center of the three canals exhibited impact waveforms. In addition, the output electric charges of the three canals were all linear to the angular acceleration, as shown in Figure 6b. The line slope of canal a# was three times that of the other two canals. This result indicated that the deformation of the cupula in the a# canal was three times that of the other two canals. The deformations of the cupula in the b# canal and c# canal were basically the same. The output electric charges of the three semicircular canals when the 3-BSC experienced a square swing around the centerline of canal b# are shown in Figure 6c,d. The results indicated that the cupula deformations in the three semicircular canals all had square waveforms, while the deformation of the cupula in canal b# was three times that in the other two canals. The output charge signals of the three semicircular canals when the 3-BSC experienced a sinusoidal swing around the centerline of the c# canal are shown in Figure 6e,f. The results indicated that the deformations of the cupula in the three semicircular canals all exhibited sinusoidal waveforms. The deformation of the cupula in the c# canal was three times that in the other two canals.

According to Ewald’s law, when the human head is rotated in the plane of one semicircular canal, the cupula in this semicircular canal will deform and cause nystagmus in this plane [30,31,32,33,34]. Do the cupulas in the other two semicircular canals also deform at the same time? Thus far, there are no relevant reports on this question. To study this problem, we carried out an angular acceleration sensing experiment on the 3-BSC. The results indicated that when one of the semicircular canals was subject to an impact swing, sinusoidal swing, or square swing around its centerline, the deformations of the cupula in it were similar to those in the 1-BSC and exhibited impact, sinusoidal, or square waveforms, respectively. The cupulas in the other two semicircular canals were also deformed, and their deformations exhibited the same waveforms but at different amplitudes than those in the main semicircular canal. The deformation of the cupula in the main semicircular canal was 3 times that of the cupula in the other two semicircular canals. This probably occurred because when the main semicircular canal rotated, the endolymph in the utricle also flowed, resulting in a change in the liquid pressure near the cupulas in the other two semicircular canals. This caused deformations in the other two cupulas; however, the deformation of these two cupulas was significantly smaller than that of the cupula in the main semicircular canal. According to the results of the experiments, when the human head is subjected to angular acceleration, the cupula in the semicircular canal will be deformed by the endolymph. The ciliary bundles of sensory hair cell in the cupula bend and send nerve signals to the superior nucleus and brain. According to the analysis of the cupula deformation of the BA in the BSC, when the head of the human body is subjected to an impact, square, or sinusoidal swing, the brain can perceive some parameters of the swing, such as the waveform, frequency, phase, and amplitude. In addition, when the human semicircular canal is in the vertical plane, gravity has a great influence on the perception of angular acceleration.

Based on these results, it is speculated that when a human head revolves around the centerline of one of the semicircular canals, the cupulas in the other two semicircular canals also deform and generate nerve signals. The human brain may obtain the specific direction and amplitude of the angular acceleration according to the relationships among the nerve signals of three semicircular canals.

At present, clinically the health status of the semicircular canal is inferred based on the VOR. According to Kim et al., when the head was under sinusoidal motion, the eyes also moved sinusoidally due to VOR [35]; that is, the movement of the eye reflex was consistent with that of the ampulla cupula. In the future, based on the patient’s nystagmus, the bionic semicircular canal fabricated in this study can be used to visually demonstrate the corresponding movement of the ampulla cupula, so that the location and pathology of the lesions, such as endolymphatic hydrops and otolithiasis, can be judged.

## 4. Conclusions

In this study, a 1-BSC and a 3-BSC were designed and fabricated to imitate the structure of the human semicircular canal. In the 1-BSC and 3-BSC, SMPFs were used to replicate the sensory hair cells in the human semicircular canal, medical silicone rubber was used to replace the polysaccharide tissue, and deionized water was used as the endolymph. Vibration testing was carried out on the 1-BSC. The results showed that when the 1-BSC was subjected to angular acceleration, the cupula could produce concave and convex deformations. When the 1-BSC was separately subjected to impact, sinusoidal, and square vibrations and the cupula also exhibited impact, sinusoidal, and square displacement, the displacement was linear with the angular acceleration. In the vertical plane, the displacement of the center of the cupula was sinusoidal with the angle of the ampullary cupula. When the 3-BSC was subjected to angular acceleration, the displacement of the center waveforms of the cupulas on these three semicircular canals in the 3-BSC were the same as for the 1-BSC. In particular, it was also found that the deformation of the BA in the main semicircular canal was exactly three times that in the other two semicircular canals. These results in this paper could provide a theoretical basis for people to better understand the working principle of the HSCs. The fact that the structure ratio is not 1:1 means the bionic model is unable to achieve absolute consistency with the human semicircular canal in terms of biomechanics. However, based on the patient’s nystagmus, the bionic semicircular canal is still promising for judging the locations and pathologies of lesions, such as endolymphatic hydrops and otolithiasis.

## Data Availability

Not applicable.

## Supplementary Materials

The following supporting information can be downloaded at: https://www.mdpi.com/article/10.3390/bioengineering9050180/s1, Figure S1: The diagram of the cross-section of the BA; Figure S2: A diagram of a bent SMPF; Figure S3: The diagram of the SMPF surface electrodes; Figure S4: The diagram of the assumed elastic structure for the membranous labyrinth of the semicircular canal; Figure S5: The stress relationship near the ampullary crest; Figure S6: The measuring system of the elastic modulus of the cupula; Figure S7: The displacement of the point of the thin film. The elastic modulus of the output charges when the bionic ampulla (BA) was deformed [1,2,36].
