# Supplementary material for: Development of Bionic Semicircular Canals and the Sensation of Angular Acceleration
Source: Bioengineering (Basel). 2022 Apr 20;9(5):180. doi: 10.3390/bioengineering9050180 (PMC9138073; doi:10.3390/bioengineering9050180)
Supplement: Supplementary file 1 [file bioengineering-09-00180-s001.zip › bioengineering-1589379-supplementary.pdf]

bioengineering

MDPI

Supplementary Materials

Development of Bionic Semicircular Canals and the Sensation of Angular Acceleration

Zhi Wang, Shien Lu, Xianjin Wang, Yuhang Chen, Junjie Gong, Yani Jiang*, Yixiang Bian*

Department of Mechanical and Electrical Engineering, College of Mechanical Engineering, Yangzhou University, Yangzhou 225012, China; laughlin222222@163.com (Z.W.); huhu1232022@163.com (S.L.) ; huyue0131@163.com (X.W.); ruyi1311@126.com (Y.C.), jjgong@yzu.edu.cn (J.G.)
* Correspondence: ynjiang@yzu.edu.cn (Y.J.); yxbian@yzu.edu.cn (Y.B.)

1. The SMPF Output Charges when the Bionic Ampulla (BA) Deformed

In the BA, an SMPF was wrapped in silicone rubber. Under the action of liquid pressure, the cupula of the BA exhibited concave and convex deformation, while the SMPF embedded within it was also bent at the same time. The silicone rubber in the BA was assumed to be a circular thin plate with a fixed periphery and with a radius of R_A, as shown in Figure S1.

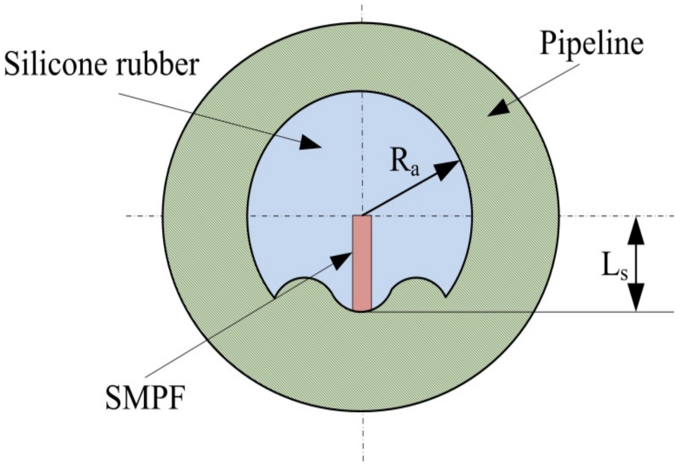

Figure S1. The diagram of the cross-section of the BA.

The semicircular canal was full of liquid, which had a pressure transmission effect. When the BA was subjected to a liquid impact force, the liquid force on the BA was assumed to be a uniform load p. The deflection curve equation of the BA at the radius of r_a was as follows:

$$w(r_a) = \frac{p}{64D'}(R_a^2 - r_a^2)^2 \tag{1}$$

where D is the bending stiffness of the silicone rubber membrane, which can be calculated as follows:

$$K = D' = \frac{Et^3}{16(1-\mu^2)} \tag{2}$$

where t represents the thickness of the silicone and μ represents Poisson's ratio.

The deflection at the central point reached the maximum, which can be described as follows:

$$w_{\max} = \frac{p}{64D'}(R_a^2 - 0)^2 = \frac{p}{64D'}R_a^4 \quad (3)$$

It can be concluded that the deflection at the center of the ampullary crest is directly proportional to the force p.

Because one end of the SMPF was fixed on the edge of the silicone rubber membrane, its axial direction was the same as the diameter direction of the silicone rubber membrane. Because the SMPF had a small volume and was easy to bend, the same deformation could occur in the SMPF at the same time as the silicone rubber was deformed. The deformation of the SMPF was equal to that of silicone rubber at the corresponding position. Taking the fixed end of the SMPF as the coordinate origin, a coordinate system was established, as shown in Figure S2.

$$w(z) = \frac{p}{64D'}(R_a^2 - (R_a - z)^2)^2 = \frac{p}{64D'}(4R_a^2z^2 - 4R_az^3 + z^4) \quad (4)$$

The curvature of any point Z at the SMPF was as follows:

$$\frac{1}{\rho(z)} = w''(z) = \frac{p}{64D'}(8R_a^2 - 24R_az + 12z^3) \quad (5)$$

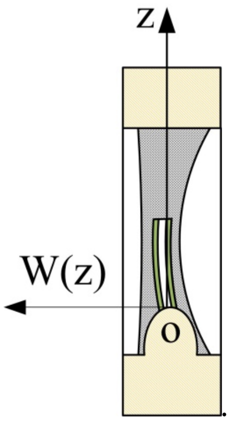

Figure S2. A diagram of a bent SMPF.

The surface strain of point Z in the SMPF coordinate can be described as follows:

$$S(z) = \frac{R_p \cos\theta}{\rho(z)} = \frac{pR_p \cos\theta}{4K}(8R_a^2 - 24R_az + 12z^2) \quad (6)$$

where R_p is the radius of the SMPF and θ is the included angle between the connecting line of any point on the surface electrode to the center of the SMPF cross-section and the median line of the electrode, with a maximum value of a_0, as shown in Figure S3.

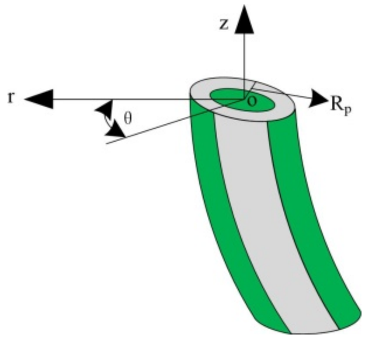

Figure S3. The diagram of the SMPF surface electrodes.

In the SMPF, the PVDF layer was a piezoelectric material. The first kind of piezoelectric equation was used to describe the piezoelectric relationship, as follows:

$$[S] = [s^E][T] + [d]^t[E] \tag{7}$$

$$[D] = [d][T] + [\varepsilon^T][E] \tag{8}$$

where S_{ij} represents the strain and T_{ij} represents the stress; $D_r = d_{31}\frac{S_{zz}}{s_{11}^E}$ represents the elastic compliance coefficient when the electric field intensity E is equal to zero or a constant; d_{ij} is the piezoelectric constant and E_i is the electric field intensity; D_i represents the electric displacement; ε_{ij}^T represents the dielectric constant when the stress T is equal to zero or a constant; the direction along the axis of the SMPF was assumed to be in the Z direction.

$$s_{rr} = s_{\theta\theta} = s_{r\theta} = s_{\theta z} = s_{rz} = 0 \tag{9}$$

$$D_\theta = D_z = 0 \tag{10}$$

$$E_r = 0 \tag{11}$$

Substituting Equations (13), (14), (14), and (15) into equations (11) and (12), the electric displacement on the SMPF electrode can be obtained as follows:

$$D_r = d_{31}\frac{S_{zz}}{s_{11}^E} \tag{12}$$

After substituting the strain S along the Z Z-directionobtained from Formula (6) into Formula (12) and integrating it, the charge on one electrode on the surface of the SMPF with length L could be obtained, as follows:

$$Q_0 = \int_0^L \int_{-\alpha_0}^{\alpha_0} d_{31}\frac{S_{zz}}{s_{11}^E}\,d\theta dx = \int_0^L \int_{-\alpha_0}^{\alpha_0} d_{31}\frac{pR_P \cos\theta}{4Ks_{11}^E}(8R^2 - 24Rx + 12x^2)\,d\theta dx \tag{13}$$

$$= \frac{pR_P \sin\alpha_0}{2Ks_{11}^E}(8R^2x - 12Rx^2 + 4x^3)_0^L = \frac{pR_P \sin\alpha_0}{2Ks_{11}^E}(8R^2L - 12RL^2 + 4L^3)$$

Because the SMPF was a completely symmetrical structure, the charges generated on the two independent electrodes on the SMPF's surface had the same value and the opposite polarity when the SMPF bending deformation occurred. The sensing signal generated on the SMPF can be described as follows:

$$Q_1 = 2Q_0 = \frac{pR_P \sin a_0}{Ks_{11}^E} = (8R^2L - 12RL^2 + 4L^3) = K_2 \quad (14)$$

It was concluded that the charge Q generated on SMPF had a linear relationship with the load P. We rewrote Formula (3) as follows:

$$p = \frac{64D'}{R_a^4} w_{max} \quad (14)$$

Substituting Formula (15) into Formula (14), we obtained the following:

$$Q_1 = K_1 w_{max} = K_2 P \quad (15)$$

It can be seen that the SMPF output charge was linear to the deflection on the central point of the BA.

2. The Deformation of Ampulla in 1-BSC Under Angular Velocity

At present, in most of the biomechanics theoretical models of the semicircular canal, the membranous labyrinth was assumed as a rigid canal with a constant internal volume. In the process of semicircular canal movement, only the inertial force of endolymph was considered. In the process of BSC rotation, the endolymph fluid in the membranous labyrinth was affected by the inertial force and friction force of the inner wall of the membranous labyrinth; for the effect of the ampullary crest, the endolymph near it deformed. The whole membranous labyrinth and the exolymph were all elastic, and the exolymph was communicated with the outer part of the semicircular canal, therefore, it is difficult to transfer the deformation to the endolymph in each part of the semicircular canal directly and rapidly and make the endolymph deform.

According to the above hypothesis, based on the anatomical structure of the human semicircular canal, we assumed the membranous labyrinth and the exolymph as an elastomer, as shown in Figure S4. The cupula of the ampulla was assumed to be a spring, and the surrounding of the cupula was fixed on the inner wall of the canal. Both ends of the spring were also fixedly connected with the liquid mass block. Because the canal was closed, the left and right liquid mass blocks were linked, that was, and their movement directions were exactly the same. When the two liquid masses slide into the canal, one side of the spring was compressed but the other side of it was stretched, which was equivalent to that two liquid masses combined into one liquid mass and acting on the spring. The mass of the combined liquid block was assumed as M, the elastic modulus of the spring was assumed as K_I, and the elastic modulus of the outer spring was assumed as K_B. Since $K_I \gg K_B$, the effect of K_B could be ignored.

In the horizontal plane, when the 1-BSC was subject to an angular velocity ω, it could not simultaneously move with the semicircular canal in a short time because the endolymph was a fluid and because the endolymph was subject to angular velocity in the opposite direction. In the circumferential direction, the force was balanced. The stress on the cupula could be described as follows:

$$p = M \times R \times \omega \tag{16}$$

Submitting Formula (17) into (16), the SMPF output charges could be obtained when the 1-BSC was subject to an angular velocity. The specific was as follows:

$$Q_2 = K_3 M R \omega \tag{17}$$

We concluded that the SMPF output charges were linear with the angular velocity ω.

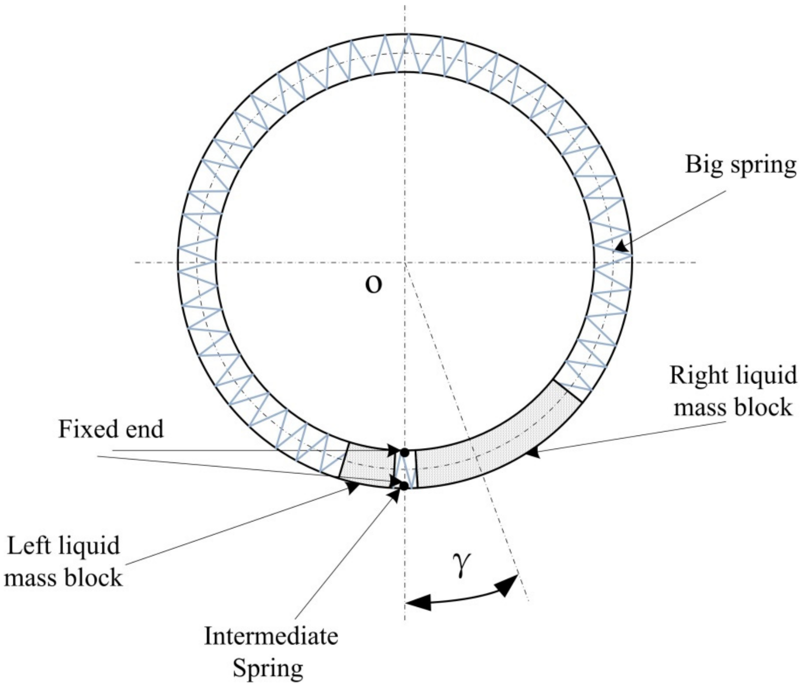

Figure S4. A diagram of the assumed elastic structure of the membranous labyrinth of the semicircular canal.

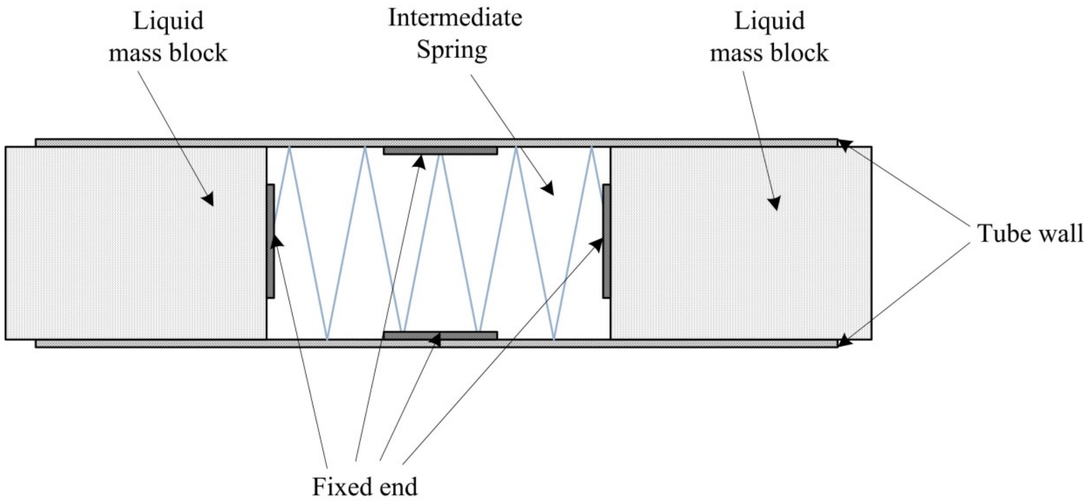

Figure S5. The stress relationship near the ampullary crest.

3. The Elastic Modulus of the Output Charges when the Bionic Ampulla (BA) Deformed

The elastic modulus of the silicone rubber in this paper was measured using the methods found in [1]. A piece of film composed of silicone rubber (E610, Shenzhen Hongye Technology Co., Ltd., Shenzhen, China.) was manufactured using the methods outlined in this paper. The circumference of the film was fixed, and after a uniform load was applied to the surface the displacement of the midpoint was measured. Substituting the external load and displacement of the film's center point into Eq. (19), the elastic modulus of the film can be calculated:

$$P = \frac{8}{3}\frac{E}{(1-v)}\frac{th^3}{a^4} \tag{18}$$

where P is the distributed load, E is the elastic modulus, v is the Poisson ratio, t is the thickness, and h is the displacement of the center point of the thin film. The measurement system is shown in Figure S6. When different loads were applied, deformations of the film occurred, as shown in Figure S7.

After experiment and calculation, the elastic modulus of the silicone rubber used in this paper was 64.12 Pa., which was more than 12 times the elastic modulus of the cupula [2].

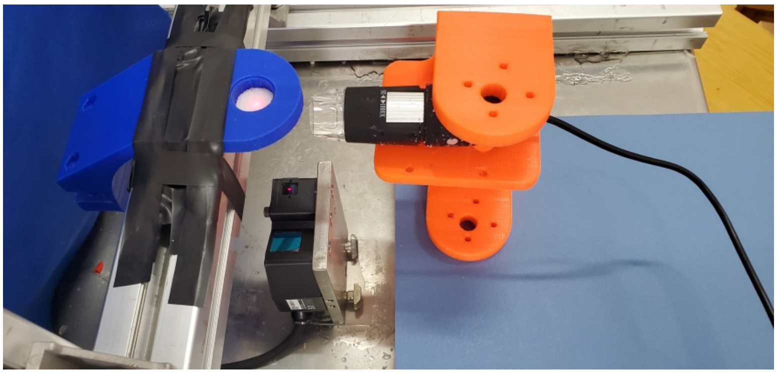

Figure S6. The measurements system used for the elastic modulus of the cupula.

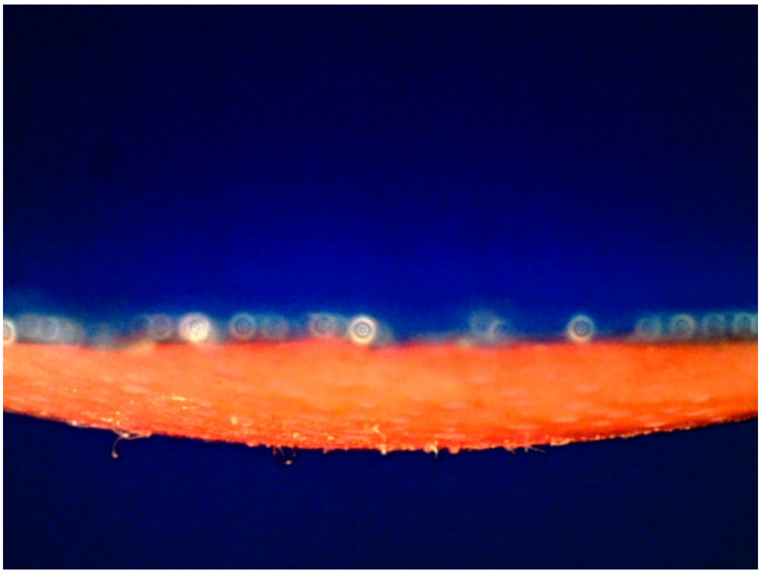

Figure S7. The displacement of the center point of thin film.

Reference

1. Neugebauer, C.A.; Newkirk, J.B.; Vermilyea, D.A.; Bauer, S.H. Structure and Properties of Thin Films. *J. Electrochem. Soc.* **1966**, *109*, 29–40.
2. Selva, P.; Oman, C.M.; Stone, H.A. Mechanical properties and motion of the cupula of the human semicircular canal. *J. Vestib. Res. Equilib. Orientat.* **2009**, *19*, 95–110.
